# A High Precision Position Sensor Design and Its Signal Processing Algorithm for a Maglev Train

**DOI:** 10.3390/s120505225

**Published:** 2012-04-26

**Authors:** Song Xue, Zhiqiang Long, Ning He, Wensen Chang

**Affiliations:** College of Mechatronics Engineering and Automation, National University of Defense Technology, Changsha, Hunan 410073, China; E-Mails: zhqlong@263.net (Z.L.); hening0606@126.com (N.H.); a_pop_song@163.com (W.C.)

**Keywords:** maglev train, high precision position sensor, tracking differentiator, signal processing, time delay compensation

## Abstract

High precision positioning technology for a kind of high speed maglev train with an electromagnetic suspension (EMS) system is studied. At first, the basic structure and functions of the position sensor are introduced and some key techniques to enhance the positioning precision are designed. Then, in order to further improve the positioning signal quality and the fault-tolerant ability of the sensor, a new kind of discrete-time tracking differentiator (TD) is proposed based on nonlinear optimal control theory. This new TD has good filtering and differentiating performances and a small calculation load. It is suitable for real-time signal processing. The stability, convergence property and frequency characteristics of the TD are studied and analyzed thoroughly. The delay constant of the TD is figured out and an effective time delay compensation algorithm is proposed. Based on the TD technology, a filtering process is introduced in to improve the positioning signal waveform when the sensor is under bad working conditions, and a two-sensor switching algorithm is designed to eliminate the positioning errors caused by the joint gaps of the long stator. The effectiveness and stability of the sensor and its signal processing algorithms are proved by the experiments on a test train during a long-term test run.

## 1. Introduction

**Figure 1.**
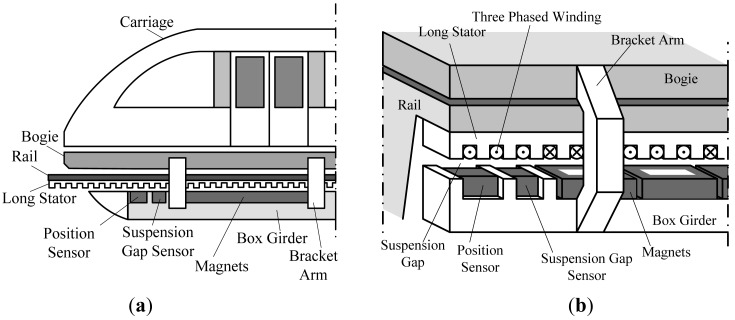
(**a**) Sketch map of high speed maglev train; (**b**) Arrangement of the long stator and the electromagnets.

The suspension function of high speed maglev trains is carried out by the electromagnetic attractive force between the electromagnets and the rail, and the train is driven by linear synchronous motor [1,2] which has high power factor and can provide strong enough tractive force. The long stator of the motor made of laminated silicon-steel sheets is installed along the rail, and the rotor is the suspension electromagnet group shown in Figure 1.

In order to reach the most efficient and stable traction performance, the traction system needs to control the current phase of the 3-phased windings to make the traveling magnetic field be synchronized with the magnetic field of the electromagnets. In this process, the precise relative position between the electromagnets and the long stator is a prerequisite. Considering the dimensional accuracy of the tooth-slot structure of the long stator, high precision positioning can be achieved by detecting the tooth-slot structure based on nondestructive detection technology [3–6].

This paper researches the system design and the signal processing algorithms of a high precision position sensor of a high speed maglev train. The sections of this paper are organized as follows: in Section 2, the operating principle of the sensor is introduced. Then, a multiple-table lookup algorithm and a suspension gap fluctuation compensation algorithm are designed to improve the positioning precision and enhance the capacity of resisting mechanical disturbances. In Section 3, the reasons for the positioning signal distortion are analyzed. In Section 4, a new kind of time-discrete tracking differentiator (TD) is proposed based on nonlinear optimal control theory. The stability, convergence property and frequency characteristics of the TD are studied and analyzed thoroughly. The delay constant of the TD is figured out, and an effective time delay compensation algorithm is designed. In Section 5, based on the TD technology, a filtering process is introduced in to improve the positioning signal waveform when the sensor is under bad working conditions, and a two-sensor switching algorithm is designed to eliminate the positioning errors caused by the joint gaps of the rails. Section 6 presents the conclusions of this paper. The effectiveness and stability of the sensor and its signal processing algorithms are proven through experiments on a test train during a long-term test run.

## 2. System and Algorithm Design for the Sensor

### 2.1. System Design

**Figure 2.**
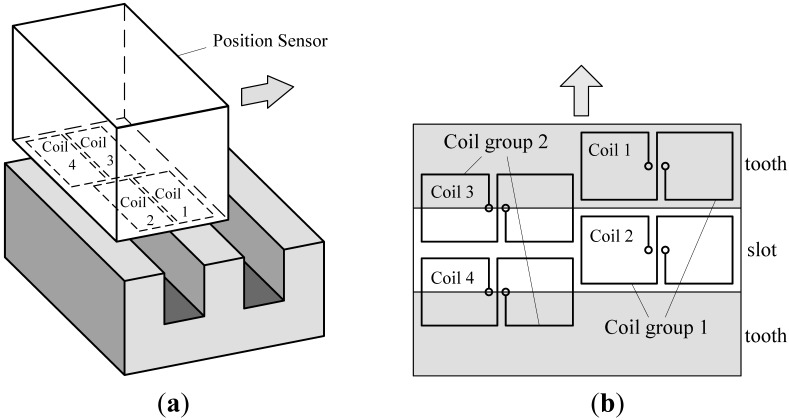
(**a**) Sketch map of the sensor; (**b**) Arrangement of the coils.

There are four same “8”-shaped coils arranged on one side of the sensor facing the long stator [7,8], shown in Figure 2.

**Figure 3.**
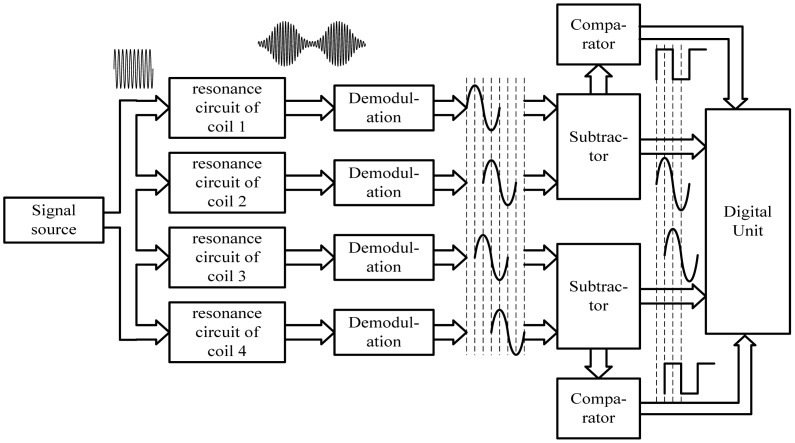
Operating principle of the sensor.

Taking one of the coils for example, the resonance circuit of the coil is stimulated by a signal source with a constant frequency. Because the electromagnetic characteristics of the long stator are different from those of the air, when the coil moves along the long stator at a certain suspension gap, its equivalent inductance changes periodically with the tooth-slot structure. Thus, the signal amplitude of the resonance circuit changes accordingly to form an amplitude-modulated signal. After demodulation, an approximated sinusoidal wave can be obtained, shown in Figure 3.

According to the relative location between the two coils in one group shown in Figure 2, the phase difference between the two demodulated signals of the two coils is 180°, shown in Figure 3. The two demodulated signals of one coil group are input into a subtractor amplifier to get their difference signal. The subtraction process can eliminate common-mode disturbances such as temperature drift. The difference signal has a better waveform and is more approximate to an ideal sine wave. According to the relative locations among the four coils, the phase difference between the two difference signals is 90°. Square waves can be obtained by putting the difference signals into comparators. The tooth-slot period number passed by the train can be gotten by counting the jumping edges of the square waves, and the phase relationship between the two square waves indicates the running direction. The phase relationship shown in Figure 3 corresponds to the situation where the sensor is moving in the direction indicated by the arrowhead shown in Figure 2.

### 2.2. Multiple-Table Lookup Algorithm

**Figure 4.**
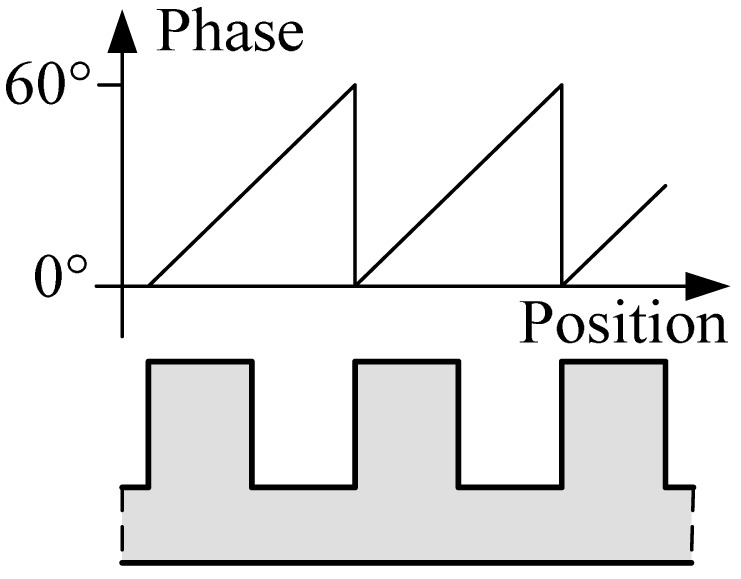
The relationship between the magnetic pole phase and the tooth-slot structure.

**Figure 5.**
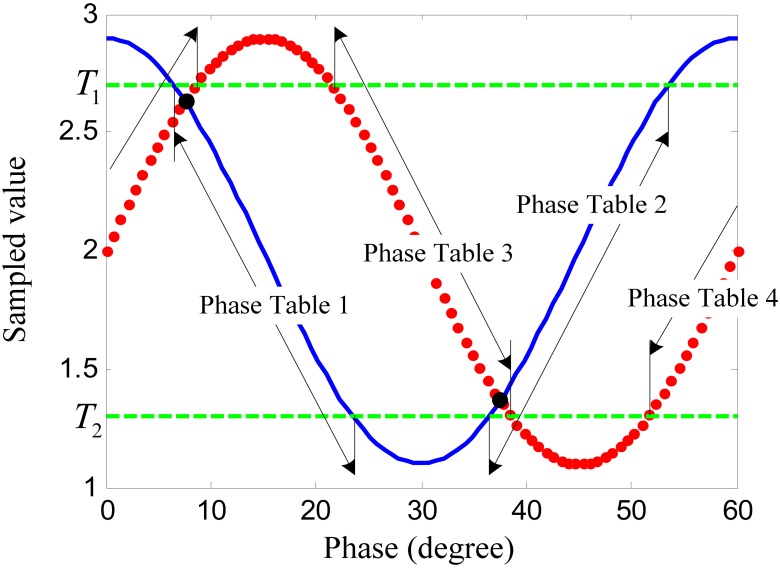
The sampled values of the difference signals in a tooth-slot period.

A magnetic pole phase period of the 3-phased windings contains six tooth-slot periods as shown in Figure 1. Thus, the length of a tooth-slot period corresponds to an electrical angle of 60° shown in Figure 4. The difference signals in a tooth-slot period are shown in Figure 5.

The data shown in Figure 5 can be used as a look-up table. Using the current sampled values of the difference signals as the indexes, the current phase can be found out from the table. However, there are some problems with the lookup phase using only one of the two sampled signals, because the signal is not monotonic and the rate of slope near the peaks and troughs is too small. Therefore, a multiple-table lookup algorithm is proposed.

At first, we choose two proper threshold values *T*_1_ and *T*_2_. *T*_1_ is slightly bigger than the upper crossing point of the two sampled signals and *T*_2_ is slightly smaller than the lower crossing point. The four signal sections between the thresholds with relatively better linearity and bigger slope rate are used as phase tables, as shown in Figure 5. In one signal processing cycle, only one phase table is chosen for phase lookup. Let *s*_1_ denote the sampled value of the difference signal of coil group 1 shown as the continuous line in Figure 5, and *s*_2_ is that of coil group 2 shown as the dotted line. If phase Table 1 or 2 is chosen in the current processing cycle, the current value of *s*_1_ is the index. Otherwise, *s*_2_ is the index. The phase table chosen in the current processing cycle is decided by the phase table chosen in the previous cycle and the relationship between the current sampled values and the threshold values. The phase table switching algorithm is shown in Table 1.

### 2.3. Suspension Gap Fluctuation Compensation Algorithm

The four phase tables are calibrated under a certain normal suspension gap. When the train is running, it's impossible for the suspension control system to make the suspension gap be absolutely invariable considering external disturbances such as the topographical relief. But the fluctuation is controlled to be within a certain range. When the suspension gap fluctuates, the amplitude and the DC components of the sampled signals deviate from the calibrated values causing considerable table lookup error. So, before the table-lookup, the sampled values should be normalized.

The latest peak value *p* and trough value *v* of the sampled signal of one coil group can be gotten by recording the sampled value at the corresponding jumping moments of the square wave of the other coil group, according to Figure 3. So, the approximate amplitude and DC component of the current sampled signal are *a* = *p* − *v* and *d* = (*p* + *v*)/2, respectively. Let *a*_0_ and *d*_0_ denote the amplitude and the DC component under the normal suspension gap and *s* denote the current sampled value. The normalized value is calculated as: *s*_0_ = (*s* − *d*)*a*_0_/*a* + *d*_0_. The influence of gap fluctuation is thus eliminated effectively by using the normalized value as the index.

## 3. Reason Analysis for Phase Signal Distortion

The sensor designed above operates well under normal working conditions, but there are still some special cases need to be considered:

### (1) Errors caused by suspension gap fluctuation compensation algorithm

The compensation algorithm supposes that the fluctuation frequency is low enough to ignore the gap change in a tooth-slot period, so the compensation is invalid for high frequency vibration, although this rarely happens. Besides, when suspension gap is too large, the amplitude of the sample signals reduces considerably. In this case, the gap fluctuation compensation method may amplify noises and errors. Further more, considering comparator hysteresis, its impossible for a jumping moment of a square wave to be exactly at the time when the sampled signal of the other coil group reaches its peaks or troughs. This also introduces errors.

### (2) Errors caused by joint gaps of long stator sections

**Figure 6.**
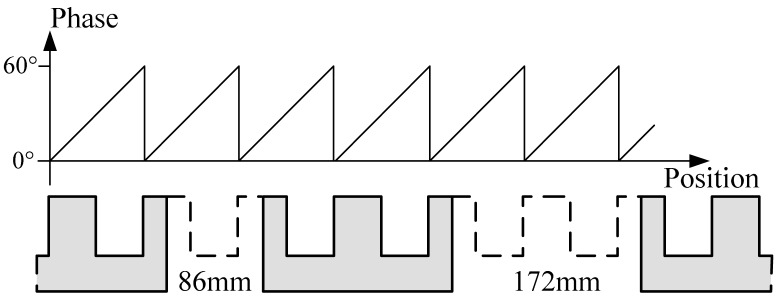
Phase waveform requirement near joint gaps.

There are two kinds of gaps with different size shown in Figure 6. The phase signal will be distorted seriously when the sensor is above a joint gap because there is no silicon-steel tooth-slot structure. But the traction system still requires normal phase signal in this situation, shown in Figure 6.

**Figure 7.**
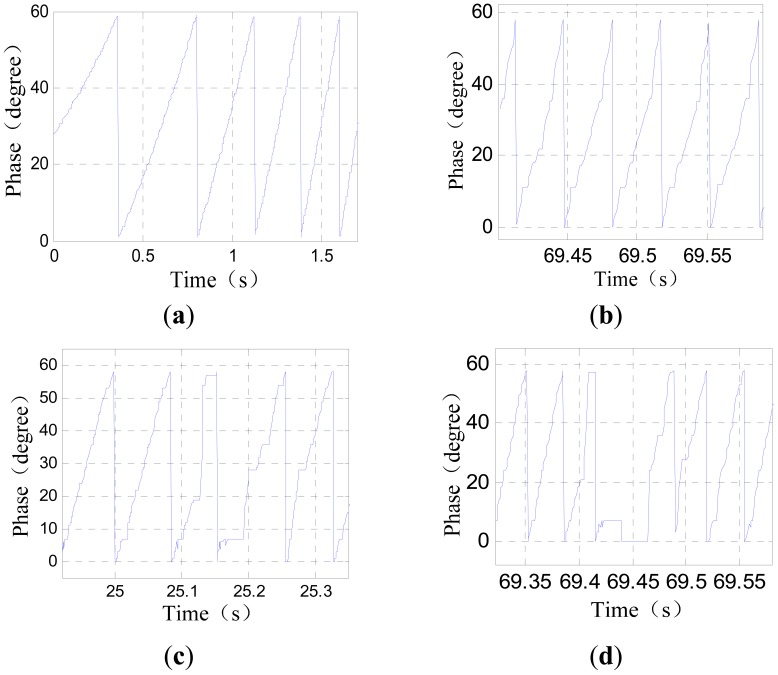
Phase data under different working conditions: (**a**) normal; (**b**) when the suspension gap is too large; (**c**) near a 86 mm joint gap; (**d**) near a 172 mm joint gap.

Figure 7 shows the phase wave data of a sensor under different working conditions. The signals are collected during a test run.

The situations shown in Figure 7(b,c) can be improved through filtering, but when the sensor passes a 172 mm joint gap as shown in Figure 7(d), tooth-slot period number counting loss happens. The tooth-slot period number obtained by the sensor is less than the number required by the traction system by 1. Thus, the phase lag is 60°. The phase nonsynchronous will reduce the efficiency of the traction and cause overcurrent protection. If the sensor has already passed several joint gaps of this size, the phase lag may become 180° causing the traction current phase to be inverted and resulting in serious accidents. Therefore, redundant position sensors are needed. Because of the structure and space limitation of the train, there are only two redundant sensors. So the switching algorithm is more complicated than that of a three-mode redundancy system. In order to solve this problem, a new kind of discrete-time tracking differentiator (TD) is proposed to further process the phase signal.

## 4. A New Kind of TD

### 4.1. Derivation of the TD

Consider the system:

(1) $$\{\begin{array}{l} {\dot{x}}_{1}=x_{2}\\ {\dot{x}}_{2}=ru,\left|u\right|\leq 1,r>0 \end{array}$$

where, *x*_1_ and *x*_2_ are the system states, *u* is the input signal and *r* is a certain constant. According to optimal control theory, the control law to drive the states of Equation (1) to the original point from any initial values in the shortest time is [9]:

(2) $$u(t)=-\text{sign}\left(x_{1}(t)+x_{2}(t)\left|x_{2}(t)\right|/2r\right)$$

Driving by input Equation (2), the states firstly move to the control law switching line:

(3) $$\Gamma (x_{1},x_{2})=x_{1}(t)+x_{2}(t)\left|x_{2}(t)\right|/2r=0$$

**Figure 8.**
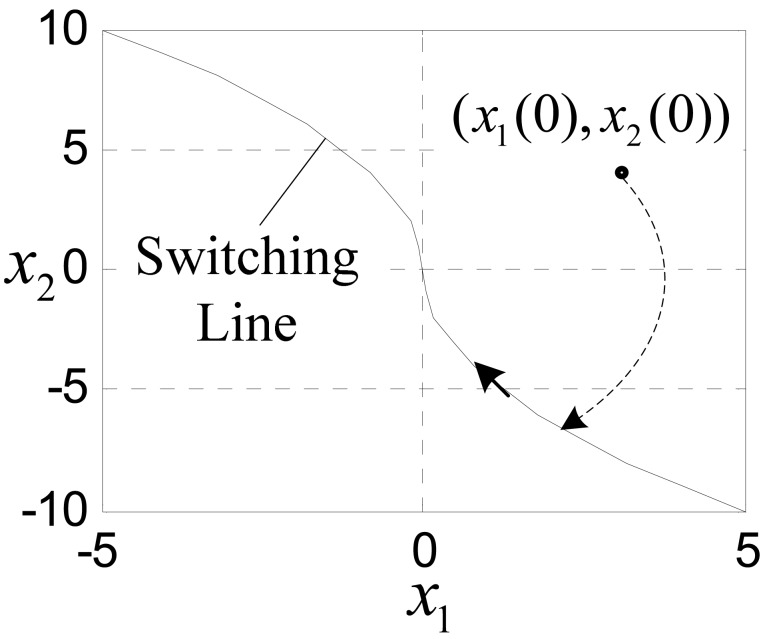
The state trajectory of Equations (1) and (2).

And then, move to the original point along the switching line, shown in Figure 8. The state trajectory shown in Figure 8 is the time optimal state trajectory.

Substituting *x*_1_(*t*) − *v*(*t*) for *x*_1_(*t*) in Equation (2), a kind of continuous-time TD is obtained as follows [10]:

(4) $$\{\begin{array}{l} {\dot{x}}_{1}=x_{2}\\ {\dot{x}}_{2}=-r\text{sign}\left(x_{1}-v+x_{2}\left|x_{2}\right|/2r\right) \end{array}$$

where, *v*(*t*) is the signal to be tracked.

For digital signal processing applications, a discrete form of Equation (4) is needed. But conventional discretization methods may bring in bad dynamic characteristics such as high frequency oscillation [10]. In order to solve this problem, [10,11] derive a discrete form of Equation (4) called as “Fhan” through Euler's polygonal arc method based on a conception of “isochronal area”. Reference [12] applies “Fhan” to zero allocation. In [13,14] the authors propose another form of discrete-time TD called “Fast” by making the state trajectory of the discrete system be coincident with that of Equation (4). In [8], research on the filtering performance of “Fast” by applying it to the phase signal of maglev train is reported. In [15] “Fast” is used to extract the derivative signal of the suspension gap sensor of a maglev train, and compare it with the integral signal of the vertical accelerometer to detect the accelerometer faults, but the two discrete forms mentioned above containing several switch conditions, have a relatively big calculation load and are not suitable for dynamic characteristic analysis. Besides, the parameter settings of the two discrete TDs are experiential. In [16,17] the authors propose another kind of nonlinear TD with high speed in whole course. Its discretization is based on Runge-Kutta-Merson method which still has relatively big calculation load.

Based on Equation (4), this paper derives another discrete TD which has a linear form but contains the meanings of nonlinear optimal control theory. The noise-resisting ability and differentiating performance of the new TD are as good, but its calculation load is relatively less, and it can be studied and analyzed using many developed theories.

By properly introduce in sampling switch and zero-order hold, a discrete system corresponding to Equation (1) is obtained as follows:

(5) $$\{\begin{array}{l} x_{1}(k+1)=x_{1}(k)+Tx_{2}(k)+eT^{2}u_{a}/2\\ x_{2}(k+1)=x_{2}(k)+eTu_{a}(k) \end{array}$$

where *T* is the discretization time step length. Usually, the states of Equation (5) need at least two steps to move to the original point from certain initial values (*x*_1_(0), *x*_2_(0)). In order to make the state trajectory of Equation (5) satisfies a certain time optimal trajectory, choose control law *u_a_*(0) satisfies:

(6) $$x_{1}(1)+\frac{1}{2r}x_{2}(1)\left|x_{2}(1)\right|=0$$

Substituting (*x*_1_(0), *x*_2_(0)) into Equation (5), and then substituting Equations (5) into (6), we have:

(7) $$u_{a}(0)=\{\begin{array}{l} \frac{-2rTx_{2}(0)-r^{2}T^{2}+rT\sqrt{r^{2}T^{2}-4r(2x_{1}(0)+Tx_{2}(0))}}{2r^{2}T^{2}},2x_{1}(0)+Tx_{2}(0)<0\\ \frac{-2rTx_{2}(0)+r^{2}T^{2}-rT\sqrt{r^{2}T^{2}+4r(2x_{1}(0)+Tx_{2}(0))}}{2r^{2}T^{2}},2x_{1}(0)+Tx_{2}(0)>0 \end{array}$$

If the initial states satisfy:

(8) $$2x_{1}(0)+Tx_{2}(0)=0$$

Let:

(9) $$u_{a}(0)=-x_{2}(0)/rT$$

Then, the system states can move to the original point in one step. Where *r* can be any nonzero value and (*x*_1_(1), *x*_2_(1)) = (0, 0).

On the other hand, to make the states of Equation (5) move to the original point in two steps, substituting (*x*_1_(0), *x*_2_(0)) into Equation (5), the expression of (*x*_1_(2), *x*_2_(2)) can be obtained. Substituting the expression into:

(10) $$\left(x_{1}(2),x_{2}(2))=(0,0\right)$$

We have:

(11) $$u_{a}(0)=-\left(2x_{1}(0)+3Tx_{2}(0)\right)/2rT^{2}$$

According to Equations (7) and (11), *r* is solved out as follows:

(12) $$r=\{\begin{array}{l} -\frac{2x_{1}(0)+Tx_{2}(0)}{2T^{2}},2x_{1}(0)+Tx_{2}(0)<0\\ \frac{2x_{1}(0)+Tx_{2}(0)}{2T^{2}},2x_{1}(0)+Tx_{2}(0)>0 \end{array}$$

If the initial values satisfy Equations (8), then (11) reduces to Equation (9), where *r* can be any nonzero value. To sum up, the discrete-time optimal control law is written as follows:

(13) $$\{\begin{array}{l} \text{if}\;2x_{1}(k)+Tx_{2}(k)\neq 0:\\ \;u_{a}(k)=-\text{sign}(2x_{1}(k)+Tx_{2}(k))\frac{2x_{1}(k)+3Tx_{2}(k)}{2x_{1}(k)+Tx_{2}(k)};\\ \;r=\text{sign}(2x_{1}(k)+Tx_{2}(k))\frac{2x_{1}(k)+Tx_{2}(k)}{2T^{2}}\\ \text{if}\;2x_{1}(k)+Tx_{2}(k)=0:\\ \;u_{a}(k)=-x_{2}(k)/rT;\\ \;r\neq 0 \end{array}$$

Furthermore, denoting *ru_a_*(*k*) as *u*(*k*), Equations (5) and (13) reduces to:

(14) $$\{\begin{array}{l} x_{1}(k+1)=x_{1}(k)+Tx_{2}(k)+T^{2}u(k)/2\\ x_{2}(k+1)=x_{2}(k)+Tu(k) \end{array}$$

(15) $$u(k)=-\left(2x_{1}(k)+3Tx_{2}(k)\right)/2T^{2}$$

Although, Equations (14) and (15) have a simple linear form, their equivalent Equations (5) and (13) reveal their physical significance in optimal control field. That is, driven by control law (15), the states of system (14) will reach a switching line determined by parameter *r*(*x*_1_(*k*), *x*_2_(*k*)) at *k* = 1, and then reach the original point at *k* = 2. Substituting *x*_1_(*k*) − *v*(*k*) for *x*_1_(*k*) and substituting *c*_0_*T* for *T* in Equation (15), we have:

(16) $$\{\begin{array}{l} x_{1}(k+1)=x_{1}(k)+Tx_{2}(k)+T^{2}u(k)/2\\ x_{2}(k+1)=x_{2}(k)+Tu(k)\\ u(k)=-\left(2(x_{1}(k)-v(k))+3c_{0}Tx_{2}(k)\right)/2c_{0}^{2}T^{2} \end{array}$$

State *x*_1_(*k*) of Equation (16) is the smoothed approximate value of signal *v*(*k*), and *x*_2_(*k*) is the approximate value of *v̇*(*k*). *c*_0_(≥1) is called as filtering factor. The bigger *c*_0_ is, the smoother *x*_1_(*k*) is, and the bigger the time delay of *x*_2_(*k*) is.

The characteristic Equation (16) is:

(17) $$2c_{0}^{2}\lambda^{2}+(-4c_{0}^{2}+3c_{0}+1)\lambda +(2c_{0}^{2}-3c_{0}+1)=0$$

It can be proved that the roots of Equation (17) satisfy ‖*λ_i_*‖ < 1, *i* = 1, 2. Thus TD (16) is stable.

**Figure 9.**
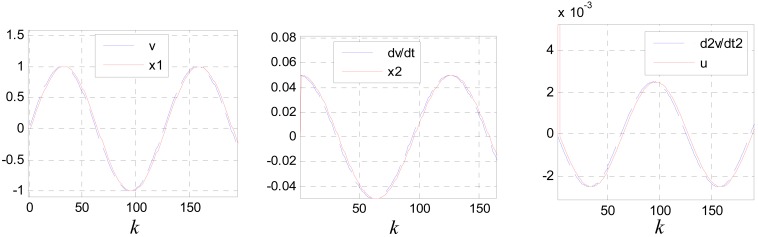
The tracking and differentiating performances of the TD designed.

Figure 9 shows the tracking and differentiating performances of the TD designed. Where: *v*(*k*) = sin(0.05*k*) and *c*_0_ = 1.

### 4.2. Convergence Property Analysis

For convenience, the analysis is carried out in continuous-time field. Consider the system:

(18) $$\{\begin{array}{l} {\dot{z}}_{1}=z_{2}\\ {\dot{z}}_{2}=f(z_{1},z_{2}) \end{array}$$

where *f*(*z*_1_, *z*_2_) = −*z*_1_ − 3*z*_2_/2. Choose the Lyapunov function as:

(19) $$V(\text{z})=17z_{1}^{2}/12+z_{1}z_{2}+2z_{2}^{2}/3$$

Its easy to prove that there exists a constant *c* > 0 satisfying:

(20) $$\dot{V}(\text{z})+cV(\text{z})\leq 0$$

Because the partial derivatives of *V*(***z***) are continuous and unbounded, *V*(***z***) satisfies local Lipschitz condition. And because of the following relationship:

(21) $$\left|f(z_{1},z_{2})-f({\tilde{z}}_{1},{\tilde{z}}_{2})\right|=\left|\left({\tilde{z}}_{1}-z_{1})+\frac{3}{2}({\tilde{z}}_{2}-z_{2}\right)\right|\leq \frac{3}{2}\left(\left|{\tilde{z}}_{1}-z_{1}\right|+\left|{\tilde{z}}_{2}-z_{2}\right|\right)$$

there exist *ñ_i_* ∈ (0, 1], *i* = 1, 2 and a nonnegative constant *A* satisfying:

(22) $$\left|f(z_{1},z_{2})-f({\tilde{z}}_{1},{\tilde{z}}_{2})\right|\leq A\sum_{i=1}^{2}{\left|{\tilde{z}}_{1}-z_{1}\right|}^{\rho_{i-1}}$$

On the other hand, consider the continuous form of Equation (16):

(23) $$\{\begin{array}{l} {\dot{x}}_{1}=x_{2}\\ {\dot{x}}_{2}=-\left(2(x_{1}-v(t))+3\varepsilon x_{2}\right)/2\varepsilon^{2} \end{array}$$

where *å* = *c*_0_*T* Though parameter setting, we can have 0 < *å* < 1. Most signals in engineering practice can be expressed by the linear combination or integral of sinusoidal signals with different frequencies. Thus, suppose *v*(*t*) is a certain sinusoidal signal. Denoting the *i*th derivative of *v*(*t*) by *v*^(^*^i^*^)^(*t*) and then denoting *x_i_* − *v*^(*i* − 1)^(*t*) by *e_i_*, the error system of Equation (23) is obtained as follows:

(24) $$\{\begin{array}{l} {\dot{e}}_{1}=e_{2}\\ \varepsilon^{2}{\dot{e}}_{2}=f(e_{1},\varepsilon e_{2}+\varepsilon \frac{\text{d}v}{\text{d}t})-\varepsilon^{1}\frac{{\text{d}}^{2}v}{\text{d}t^{2}} \end{array}$$

Let *ô* = *t*/*å, z*_1_(*ô*) = *e*_1_(*t*), *z*_2_(*ô*) = *åe*_2_(*t*), then Equation (24) is converted to [18]:

(25) $$\{\begin{array}{l} \frac{\text{d}z_{1}}{\text{d}\tau }=z_{2}\\ \frac{\text{d}z_{2}}{\text{d}\tau }=f(z_{1},z_{2}+\frac{\text{d}v}{\text{d}\tau })-\frac{{\text{d}}^{2}v}{\text{d}\tau^{2}} \end{array}$$

Choose Lyapnov function as (*V* ∘ ***z***)(*ô*). Because (*V****z***) is locally Lipschtiz continuous, (*V* ∘***z***)(*ô*) is locally Lipschtiz continuous too. Considering most signals in engineering practice are bounded, and it is easy to prove that Equation (23) is stable, so, ***z***(*ô*) is bounded. Thus, supposing the Lipschitz constant of (*V* ∘***z***)(*ô*) is *M*, we have [18]:

(26) $$\begin{array}{ll} D^{+}(V\circ \text{z})(\tau ) & =\frac{\partial V(\text{z})}{\partial \text{z}}{\left[\begin{array}{ll} z_{2} & f(z_{1},z_{2}+\frac{\text{d}v}{\text{d}\tau })-\frac{{\text{d}}^{2}v}{\text{d}\tau^{2}} \end{array}\right]}^{\text{T}}\\ & =\frac{\partial V(\text{z})}{\partial \text{z}}{\left[\begin{array}{ll} z_{2} & f(z_{1},z_{2}) \end{array}\right]}^{\text{T}}+\frac{\partial V(\text{z})}{\partial \text{z}}{\left[\begin{array}{ll} z_{2} & f(z_{1},z_{2}+\frac{\text{d}v}{\text{d}\tau })-\frac{{\text{d}}^{2}v}{\text{d}\tau^{2}} \end{array}\right]}^{\text{T}}-\frac{\partial V(\text{z})}{\partial \text{z}}{\left[\begin{array}{ll} z_{2} & f(z_{1},z_{2}) \end{array}\right]}^{\text{T}}\\ & \leq \dot{V}+MA{\left|\frac{\text{d}v}{\text{d}\tau }\right|}^{\rho_{1}}+M\left|\frac{{\text{d}}^{2}v}{\text{d}\tau^{2}}\right|=\dot{V}+M\left(A\varepsilon^{\rho_{1}}{\left|\frac{\text{d}v}{\text{d}t}\right|}^{\rho_{1}}+\varepsilon^{2}\left|\frac{{\text{d}}^{2}v}{\text{d}t^{2}}\right|\right)\\ & \leq \dot{V}+M\left(Al_{1}^{\rho_{1}}\varepsilon^{\rho_{1}}+l_{2}\varepsilon^{2}\right)\leq \dot{V}+\varepsilon^{\rho_{1}}M\left(Al_{1}^{\rho_{1}}+l_{2}\varepsilon^{2}\right)\\ & =\dot{V}+\varepsilon^{\rho_{1}}M\delta \end{array}$$

where *D*^+^(*V* ∘***z***)(*ô*) is the right upper derivative [19] of (*V* ∘***z***)(*ô*), *l*_1_ and *l*_2_ are the upper bounds of the first derivative and second derivative of *v*(*t*) respectively. $\ddot{a}=Al_{1}^{\rho_{1}}+l_{2}$. When *ô* ≥ *ç*, there exist constants *ç* > 0 and *r*_0_ > 0 satisfying [19]:

(27) $$\left\|\text{z}(\tau )\right\|\leq \frac{{\left(V(\text{z}(\tau ))\right)}^{1-\theta }}{r_{0}c(1-\theta )}\leq \frac{{\left(\frac{2\varepsilon^{\rho_{1}}M\delta }{c}\right)}^{\frac{1-\theta }{\theta }}}{r_{0}c(1-\theta )}$$

where *θ* ∈ (0, min(*ρ*_1_/(*ρ*_1_+2), 1/2)). Let *ε*′ = min ((*c*/(2*Mδ*))^1/*ρ*_1_^, 1) and *ε* ∈ (0,*ε*′), *l* = 1/(*r*_0_*c*(1 − *θ*)), *μ* = 2*Mδ*/*c*, and *γ* = (1 − *θ*)/*θ*. When *ô* ≥ *åç*, we have [18]:

(28) $$\left\|{\left[e_{1},\varepsilon e_{2}\right]}^{\text{T}}\right\|\leq \varepsilon^{\rho_{1}\gamma }l\mu^{\gamma }\Rightarrow \left|e_{i}\right|\leq \varepsilon^{\rho_{1}\gamma -i+1}l\mu^{\gamma },i=1,2$$

Thus:

(29) $$\left|x_{i}-v^{\left(i-1\right)}(t)\right|=O(\varepsilon^{\rho_{1}\gamma -i+1}),i=1,2$$

Because *è* can be chosen to be small enough, considering *è* < *ñ*_1_(*ñ*_1_ + 2), we have: *ñ*_1_(1 − *è*)/*è* − 2 = *ñ*_1_*ã* − 2 > 0. Thus, *ñ*_1_*ã* − *i* + 1> 1 (*i* = 1, 2) [18,19].

### 4.3. Frequency Characteristic Analysis

**Figure 10.**
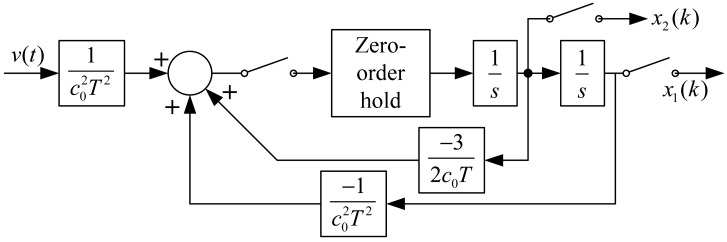
Block diagram of Equation (16).

The block diagram of Equation (16) is shown as Figure 10.

Its transfer function is:

(30) $$T_{f}(z)=\frac{x_{1}(z)}{v(z)}=\frac{z+1}{2c_{0}^{2}z^{2}+(-4c_{0}^{2}+3c_{0}+1)z+(2c_{0}^{2}-3c_{0}+1)}$$

Substitute z = e*^jωT^* = cos(*ωT*) + *j* sin(*ωT*) into Equation (30), we have:

(31) $$T_{f}(\omega )=\frac{R_{e}(\omega )+jI_{m}(\omega )}{D_{m}(\omega )}$$

where:

(32) $$R_{e}(\omega )=2\cos(\omega T)+4c_{0}^{2}\cos^{2}(\omega T)-4c_{0}^{2}+2$$

(33) $$I_{m}(\omega )=(4c_{0}^{2}-6c_{0})\sin(\omega T)-4c_{0}^{2}\sin(\omega T)\cos(\omega T)$$

(34) $$D_{m}(\omega )={\left(2c_{0}^{2}\cos(2\omega T)+(-4c_{0}^{2}+3c_{0}+1)\cos(\omega T)+(2c_{0}^{2}-3c_{0}+1)\right)}^{2}+{\left(2c_{0}^{2}\sin(2\omega T)+(-4c_{0}^{2}+3c_{0}+1)\sin(\omega T)\right)}^{2}$$

**Figure 11.**
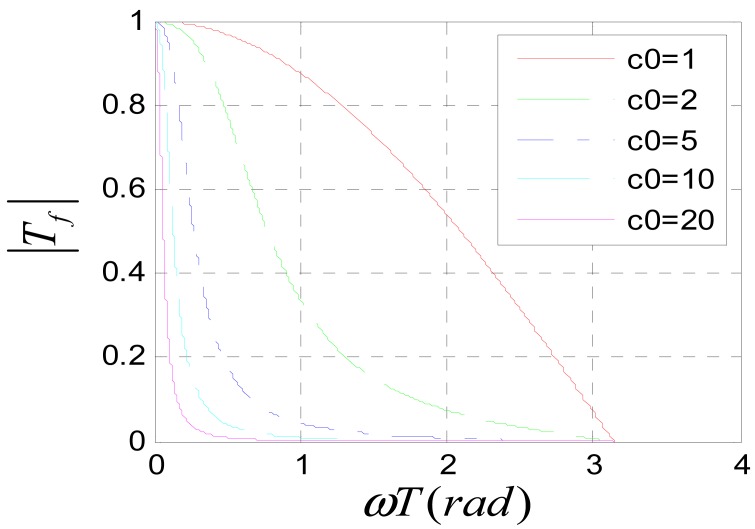
Amplitude-frequency characteristic of Equation (31).

**Figure 12.**
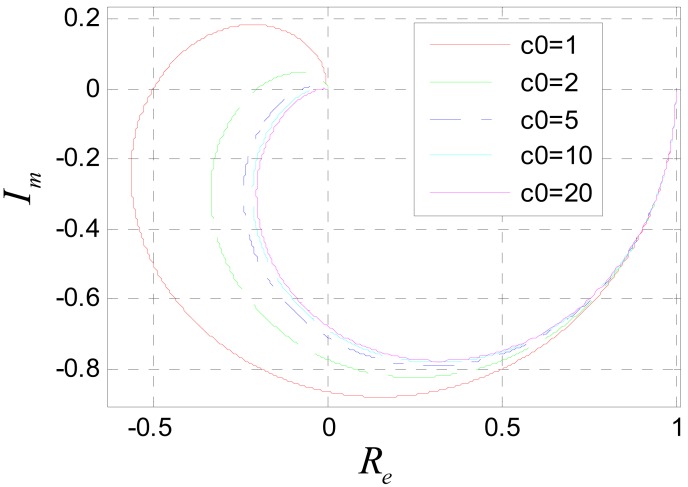
Nyquist diagram of Equation (31).

To avoid frequency aliasing, according to Shannon's sampling theorem, it should be satisfied that *ùT* < *ðrad*. So only the frequency characteristics under the condition *ùT* < *ðrad* are studied. Figure 11 shows the amplitude-frequency characteristic of system (31) when *c*_0_ is assigned to different values. It can be seen that the system is a low pass filter. The bigger *c*_0_ is, the lower the pass band is. According to the amplitude-frequency characteristic diagram, to make the amplitude of the filtered signal be approximate to that of the original signal, it should be satisfied that *c*_0_*ùT* ≪ *ðrad*. Usually, the position signal of maglev train satisfies this requirement. Figure 12 is the Nyquist diagram of Equation (31).

The phase-frequency characteristic of Equation (31) is:

(35) $$∠T_{f}(\omega )=\arctan\left(\frac{I_{m}(\omega )}{R_{e}(\omega )}\right)=\arctan\left(\frac{\left(4c_{0}^{2}-6c_{0})\sin(\omega T)-4c_{0}^{2}\sin(\omega T)\cos(\omega T\right)}{2\cos(\omega T)+4c_{0}^{2}\cos^{2}(\omega T)-4c_{0}^{2}+3}\right)$$

where, ∠*T_f_*(*ω*) is the phase angle of complex function *T_f_*(*ù*). When *c*_0_*ùT* ≪ *ðrad, I_m_*(*ù*)/*R_e_*(*ù*) can be approximated by its first order Taylor expansion as follows:

(36) $${\frac{I_{m}(\omega )}{R_{e}(\omega )}\approx \frac{I_{m}(0)}{R_{e}(0)}+{\left(\frac{I_{m}(\omega )}{R_{e}(\omega )}\right)}^{\prime }|}_{\omega =0}{\omega =-\frac{3}{2}c_{0}T\omega =\tan(0)+{\left(\tan(\omega )\right)}^{\prime }|}_{\omega =0}\omega \approx \tan(-\frac{3}{2}c_{0}T\omega )$$

Thus:

(37) $$∠T_{f}(\omega )\approx -\frac{3}{2}c_{0}T\omega$$

Considering that a phase lag of *ù* is equivalent to a time lag of 1/*ù* second, the time lag constant of Equation (31) is calculated as:

(38) $$\tau =∠T_{f}(\omega )\frac{1}{\omega }\approx -\frac{3}{2}c_{0}T$$

**Figure 13.**
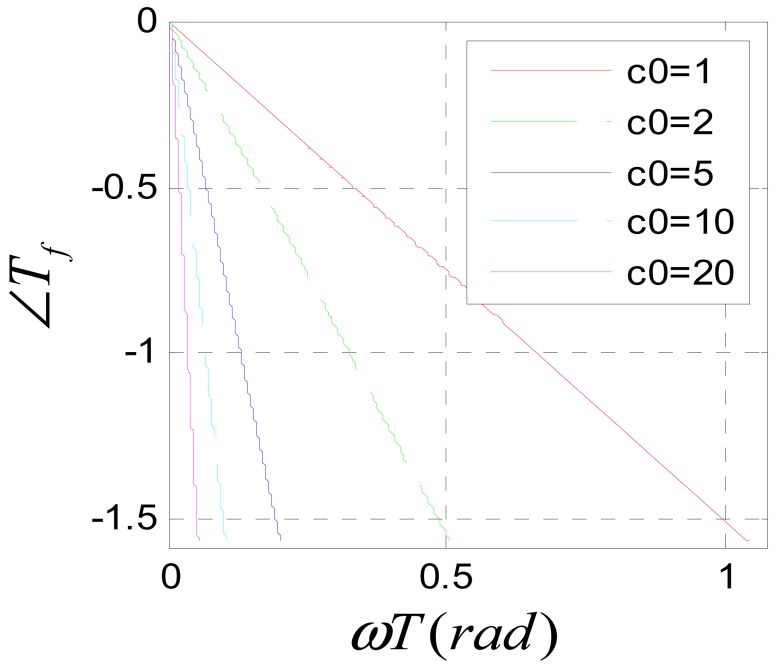
Phase-frequency characteristic of Equation (31).

Figure 13 is the phase-frequency characteristic diagram of Equation (31). It can be seen, when *c*_0_*ωT* ≪ *πrad*, the phase-frequency characteristic function is approximated to a linear function of *ω*.

### 4.4. Time Delay Compensation

**Figure 14.**
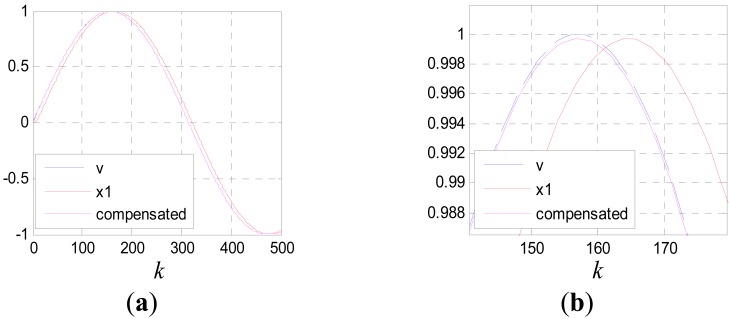
(**a**) Compensation effect for *x*_1_(*k*); (**b**) partial enlarged drawing of (a).

**Figure 15.**
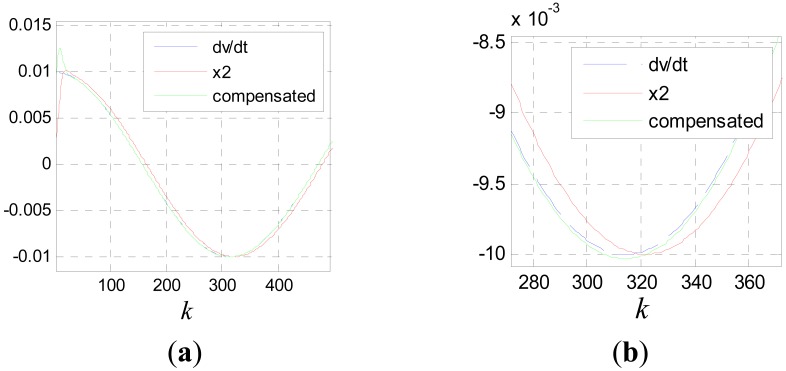
(**a**) Compensation effect for *x*_2_(*k*); (**b**) partial enlarged drawing of (a).

Maglev trains' acceleration and change rate are limited to a relatively small range, so they can be approximately treated as constants in time span *ô*. Thus, we have the following compensation algorithm:

(39) $$v(t)\approx v(t-\tau )+\dot{v}(t-\tau )\tau +\frac{1}{2}\ddot{v}(t-\tau )\tau^{2}$$

(40) $$\dot{v}(t)\approx \dot{v}(t-\tau )+\ddot{v}(t-\tau )\tau$$

where, *u*(*kT*) ≈ *v̈*(*kT* − *τ*). The compensation effect is shown in Figures 14 and 15. Where, the input signal is *v*(*k*) = sin(0.01*k*) and *c*_0_ = 5.

If high order compensation is needed, multiple tracking differentiators should be combined. Reference [8] proposes a time delay compensation algorithm for the situation where *ô* is unknown. In this paper, *ô* is figured out approximately, so the compensation is more flexible. According to a Taylor formula, we have:

(41) $$\left[\begin{array}{c} v(t-\tau )\\ \dot{v}(t-\tau )\\ \ddot{v}(t-\tau )\\ v(t-2\tau )\\ \dot{v}(t-2\tau )\\ \ddot{v}(t-2\tau ) \end{array}\right]\approx \left[\begin{array}{cccccc} 1 & -\tau & \tau^{2}/2 & -\tau^{3}/6 & \tau^{4}/24 & -\tau^{5}/120\\ 0 & 1 & -\tau & \tau^{2}/2 & -\tau^{3}/6 & \tau^{4}/24\\ 0 & 0 & 1 & -\tau & \tau^{2}/2 & -\tau^{3}/6\\ 1 & -2\tau & 2\tau^{2} & -4\tau^{3}/3 & 2t^{4}/3 & -4\tau^{5}/15\\ 0 & 1 & -2\tau & 2\tau^{2} & -4\tau^{3}/3 & 2\tau^{4}/3\\ 0 & 0 & 1 & -2\tau & 2\tau^{2} & -4\tau^{3}/3 \end{array}\right]\;\left[\begin{array}{c} v(t)\\ \dot{v}(t)\\ \ddot{v}(t)\\ \dddot{v}(t)\\ v^{\left(4\right)}(t)\\ v^{\left(5\right)}(t) \end{array}\right]$$

Equation (41) can be converted to:

(42) $$\left[\begin{array}{c} v(t-\tau )\\ \tau \dot{v}(t-\tau )\\ \tau^{2}\ddot{v}(t-\tau )\\ v(t-2\tau )\\ \tau \dot{v}(t-2\tau )\\ \tau^{2}\ddot{v}(t-2\tau ) \end{array}\right]\approx \left[\begin{array}{cccccc} 1 & -1 & 1/2 & -1/6 & 1/24 & -1/120\\ 0 & 1 & -1 & 1/2 & -1/6 & 1/24\\ 0 & 0 & 1 & -1 & 1/2 & -1/6\\ 1 & -2 & 2 & -4/3 & 2/3 & -4/15\\ 0 & 1 & -2 & 2 & -4/3 & 2/3\\ 0 & 0 & 1 & -2 & 2 & -4/3 \end{array}\right]\;\left[\begin{array}{c} v(t)\\ \tau \dot{v}(t)\\ \tau^{2}\ddot{v}(t)\\ \tau^{3}\dddot{v}(t)\\ \tau^{4}v^{\left(4\right)}(t)\\ \tau^{5}v^{\left(5\right)}(t) \end{array}\right]$$

Inverting the matrix in Equation (42), we have:

(43) $$\left[\begin{array}{c} v(t)\\ \tau \dot{v}(t)\\ \tau^{2}\ddot{v}(t)\\ \tau^{3}\dddot{v}(t)\\ \tau^{4}v^{\left(4\right)}(t)\\ \tau^{5}v^{\left(5\right)}(t) \end{array}\right]\approx \left[\begin{array}{cccccc} 32 & -16 & 4 & -31 & -14 & -2\\ 120 & -64 & 14 & -120 & -55 & -8\\ 360 & -192 & 38 & -360 & -168 & -25\\ 780 & -408 & 75 & -780 & -372 & -57\\ 1080 & -552 & 96 & -1080 & -528 & -84\\ 720 & -360 & 60 & -720 & -360 & -60 \end{array}\right]\;\left[\begin{array}{c} v(t-\tau )\\ \tau \dot{v}(t-\tau )\\ \tau^{2}\ddot{v}(t-\tau )\\ v(t-2\tau )\\ \tau \dot{v}(t-2\tau )\\ \tau^{2}\ddot{v}(t-2\tau ) \end{array}\right]$$

Thus:

(44) $$v(t)\approx 32v(t-\tau )-16\tau \dot{v}(t-\tau )+4\tau^{2}\ddot{v}(t-\tau )-31v(t-2\tau )-14\tau \dot{v}(t-2\tau )-2\tau^{2}\ddot{v}(t-2\tau )$$

**Figure 16.**
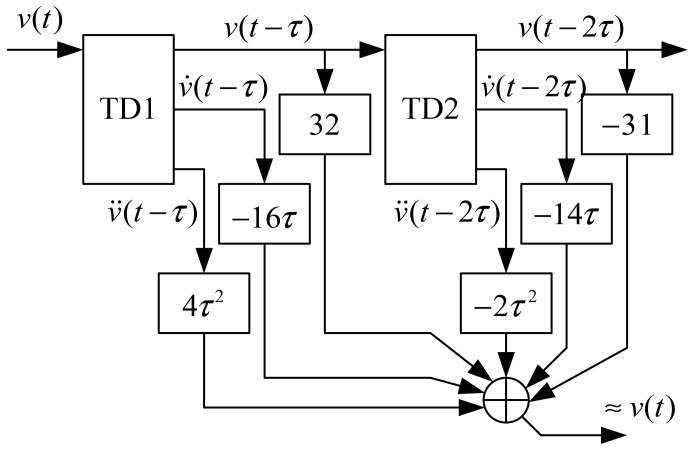
Block diagram of compensation Equation (44).

The block diagram of compensation Equation (44) is shown in Figure 16. By the same method, more tracking differentiators can be added to the algorithm to realize higher order compensation.

**Figure 17.**
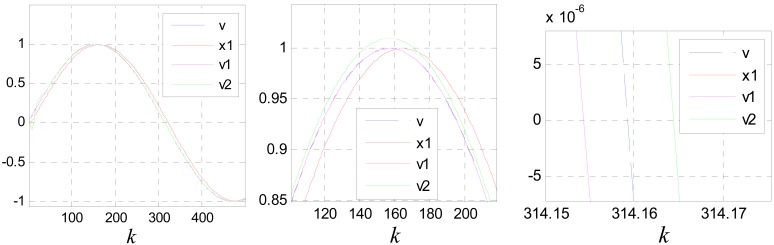
Compensation effect diagram for *x*_1_(*k*) with different scale.

Figure 17 shows the compensation effect, where, *v*_1_ and *v*_2_ are compensation results of Equations (39) and (44) respectively.

**Figure 18.**
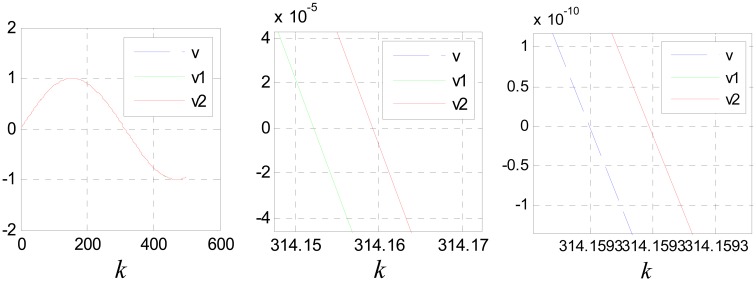
Compensation effect diagram for *x*_1_(*k*) with different scale when *ô* is known precisely.

It can be seen that the performance of Equation (39) is better than that of Equation (44). This is because Equation (44) is more sensitive to the precision of *ô*. The estimation error of *ô* will be cumulated and magnified though multi-stage filtering. But if *ô* is known precisely, the performance of Equation (44) is much better than that of Equation (39). Figure 18 shows the compensation effect when *ô* is known precisely.

**Figure 19.**
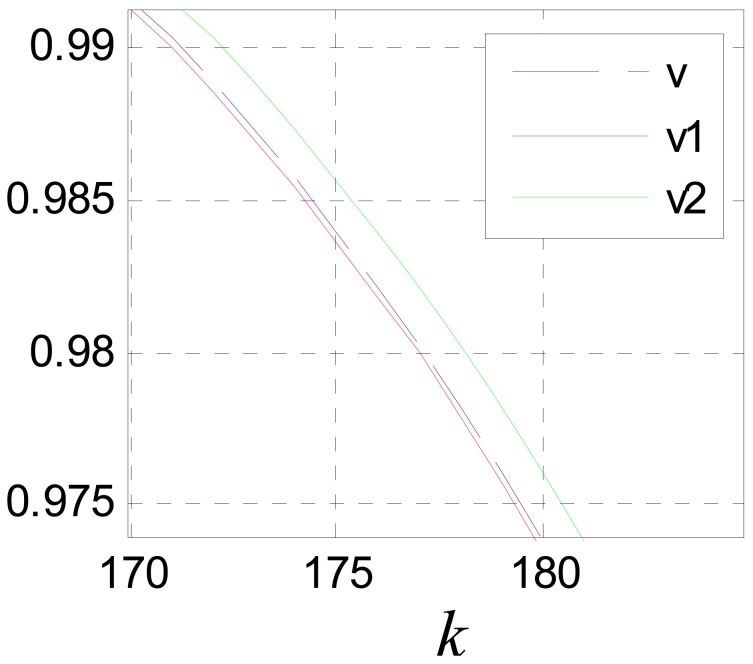
Compensation effect diagram for *x*_1_(*k*).

The compensation method proposed in [8] implies that the time delays of each filtering stage are the same which is not satisfied exactly, so its compensation effect is also relatively worse. Figure 19 shows the compensation effect of the method proposed in reference [8], where *v*_1_ and *v*_2_ are compensation results of Equation (39) and the algorithm mentioned in [8].

## 5. Experimental Results

### 5.1. Experiment Platform

The experiments are carried out on the 1.5 km test line in Shanghai. The position sensors designed are installed in the box girder of a maglev train shown in Figure 1. The sensors are connected to an onboard computer via a communication cable. Once a sensor figure out a phase datum, it is sent to the computer immediately. The computer further processes the data according to the algorithms designed in the later sections, and then sends the final results to the traction system.

The onboard computer can save and monitor the original data, midcourse data and final data on-line, and the saved data can be analyzed off-line to guide the algorithm modification and program debugging. In the debugging phase, the train is pulled by a rail test truck. After the performance of the positioning system is verified to some extent, traction system can be introduced in to drive the train and further test the positioning system. Generally, the performance of the positioning system can be verified in several ways:

1. The slope edges of the saw-tooth phase signal wave is always fine and smooth in any case as shown in Figure 7(a).
2. There are flags located on certain points on the rail encode with data indicating the precise position of these points. When the train passes a flag, a special onboard instrument can read out the precise position information encoded on the flag and the send it to the onboard computer. With this information, the computer can check the validity of the position sensor.
3. If the phase signal is incorrect, the traction efficiency will be reduced. The traction efficiency can be estimated according to the traction current and the train's velocity and acceleration.

However, considering the text length, only the experiment results when the positioning system is under certain bad working conditions are given in this paper.

### 5.2. Filtering Results

Taking the data shown in Figure 7(c) as an example, the jumping edges of the phase signal contain high frequency components. Filtering the phase signal straightforwardly will cause a serious information loss, so at first, the phase signal needs to be converted to a certain form suitable for filtering. Let *ph*(*k*) denote the current phase value and *n*(*k*) denote the current tooth-slot period number. Considering a tooth-slot period is corresponding to a phase angle of 60°, the converted signal is calculated as follows:

(45) $$p_{ha}(k)=60n(k)+p_{h}(k)$$

**Figure 20.**
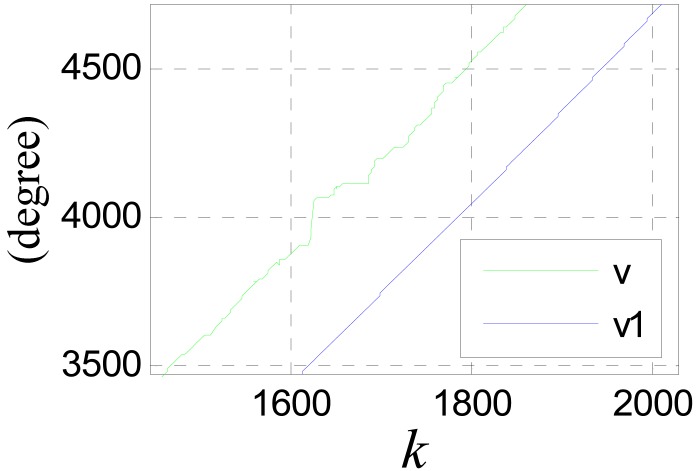
Filtering effect of the phase signal.

Because of the slight time difference between the jumping edges of the phase signal and those of the tooth-slot number signal, there are spike pulses in signal *ph_a_*(*k*). The spike pulses can be eliminated though simple logical pretreatment. Figure 20 shows the filtering effect without time delay compensation, where *c*_0_ = 100, *T* = 0.001*s, v* is the pretreated signal *ph_a_*(*k*) and *v*_1_ is the filtered signal.

**Figure 21.**
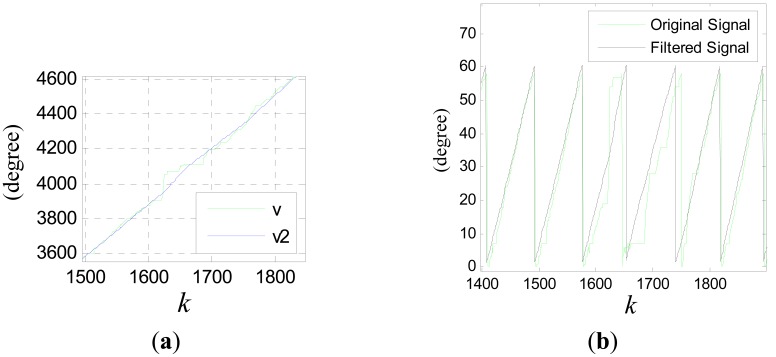
(**a**) Compensated signal (**b**) Final processing result of the phase signal.

It can be seen that the smoothing effect is good, but the time delay is serious which will result in considerable positioning errors. Figure 21 shows the results after time delay compensation, where *v*_2_ is the compensated signal. It can be seen, the phase waveform is improved obviously after the procedure.

### 5.3. Switching Experiment Results

**Figure 22.**
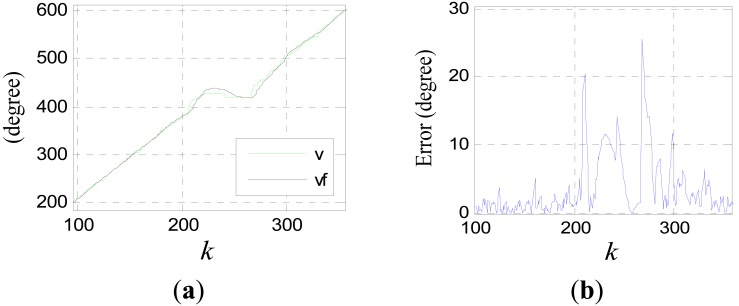
(**a**) Forecasted effect (**b**) error signal.

Denote the compensated differential signal and filtered signal of *v*(*k*) by *ṽ̇*(*k*) and *ṽ*(*k*), respectively. Forecasting *v*(*k* + 1) by *ṽ*(*k*) and *ṽ̇*(*k*), the error between the forecasted value and the real value can be obtained as:

(46) $$e(k+1)=\left|v(k+1)-v_{f}(k+1)\right|$$

where *v_f_*(*k*+1) = *ṽ*(*k*) + *ṽ̇*(*k*)*T*. When the error exceeds a certain threshold value, it can be regard that the sensor is passing a joint gap, so the switching is triggered at this moment. Figure 22 shows the forecast signal and the error signal of the data shown in Figure 7(d), where *v* is the pretreated signal *ph_a_*(*k*), and *v_f_* is the forecast signal. Setting the threshold value to be 10, Switching can be triggered at *k* = 208.

**Figure 23.**
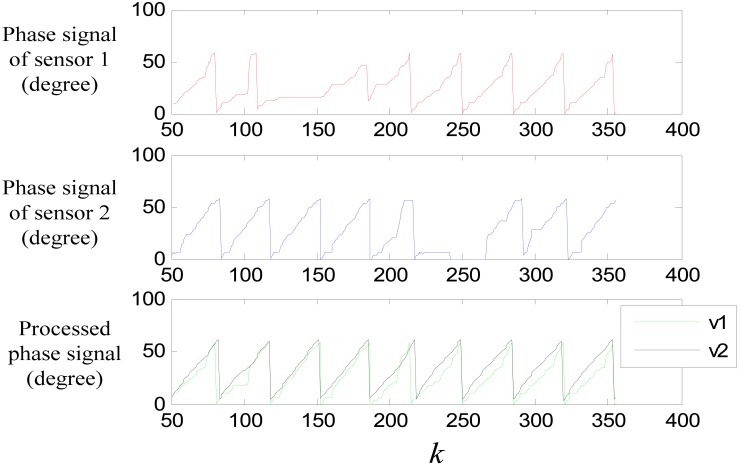
Switching experiment result.

Figure 23 shows the performance of the switching algorithm, where *v*_1_ is the combined signal of sensor 1 and sensor 2 though the switching algorithm, and *v*_2_ is the filtered signal. It can be seen that, the algorithm guaranties a normal signal when the sensors are passing a large joint gap, and tooth-slot period counting loss is avoided.

## 6. Conclusions

High precision positioning technology for a high speed maglev train is studied. At first, the operating principle of the position sensor is introduced. A multiple-table lookup algorithm is proposed to improve the linearity of the phase table and the table-lookup precision. A suspension gap fluctuation compensation method is designed to enhance the sensor's capacity of resisting mechanical disturbance. Then, in order to further improve the signal waveform and enhance the reliability of the sensor, a new kind of discrete tracking differentiator is proposed to do some further signal processing based on optimal control theory. The new TD has good filtering and differentiating performances and relatively small calculation load. The dynamic characteristics of the TD are studied thoroughly. The delay constant of the TD is figured out and an effective time delay compensation algorithm is proposed. The TD is use to filter the phase signal, obviously improving the waveform. Finally, a two-sensor switching algorithm is designed based on the TD to avoid the phase signal distortion and tooth-slot period counting loss caused by the large joint gap. The designed sensor performed well during long-term testing runs.

## Figures and Tables

**Table 1.** Phase table switching algorithm.

**Phase table used in the previous cycle**	**Relationship between the current sampled values and the threshold values**	**Phase table chosen in the current cycle**
Phase Table 1 or 2	*s*_1_ > *T*_1_	Phase Table 4
	*s*_1_ < *T*_2_	Phase Table 3
	*T*_2_ ≤ *s*_1_ < *T*_1_	No switching
Phase Table 3 or 4	*s*_2_ > *T*_1_	Phase Table 1
	*s*_2_ < *T*_2_	Phase Table 2
	*T*_2_ ≤ *s*_2_ < *T*_1_	No switching

## References

[1] Liu H.Q. (1995). Transrapid.

[2] Wu X.M. (2003). Maglev Train.

[3] Deng Y., Liu X. (2011). Electromagnetic imaging methods for nondestructive evaluation applications. Sensors.

[4] Javier G.-M., Jaime G.-G., Ernesto V.-S. (2011). Non-destructive techniques based on eddy current testing. Sensors.

[5] Zhu Y.-K., Tian G.-Y., Lu R.-S., Zhang H. (2011). A review of optical NDT technologies. Sensors.

[6] Abdelhalim Z., Hocine M., Mouloud F., Gérard B. (2010). Inverse problem in nondestructive testing using arrayed eddy current sensors. Sensors.

[7] Song X., Dai C., Long Z. Research on location and speed detection for high speed maglev train based on long stator.

[8] Dai C., Long Z., Xie Y., Song X. (2011). Research on the filtering algorithm in speed and position detection of maglev trains. Sensors.

[9] Zhong Q. (2004). Modern Control Theory.

[10] Han J.Q. (2008). Active Disturbance Rejection Control Technique—The Technique for Estimating and Compensating the Uncertainties.

[11] Han J.Q. (2009). From PID to active disturbance rejection control. IEEE Trans. Ind. Electron..

[12] Zhang W., Han J. (2001). The application of tracking differentiator in allocation of zero. Acta Autom. Sin..

[13] Xie Y.D., Long Z.Q. (2009). A high-speed nonlinear discrete tracking-differentiator with high precision. Control Theory Appl..

[14] Xie Y.D., Long Z.Q., Li J., Zhang K., Luo K. Research on a new nonlinear discrete-time tracking-differentiator filtering characteristic.

[15] Long Z., Song X., He G., Xie Y. (2011). Fault-diagnose for the accelerometer of suspension system based on signal compare. Chin. J. Sci. Instrum..

[16] Wang X., Chen Z., Yuan Z. (2003). Nonlinear tracking-differentiator with high speed in whole course. Control Theory Appl..

[17] Wang X., Chen Z., Yuan Z. (2002). Analyse and improvement for nonlinear tracking-dfferentiator. Control Decis..

[18] Wang X., Liu J. (2010). Differentiator Design and Application.

[19] Bhat S.P., Bernstein D.S. (2000). Finite-time stability of continuous autonomous system. SIAM J. Control Optim..

